# Critical role of *OX40* in the expansion and survival of CD4 T-cell-derived double-negative T cells

**DOI:** 10.1038/s41419-018-0659-x

**Published:** 2018-05-23

**Authors:** Guangyong Sun, Xiaojing Sun, Wei Li, Kai Liu, Dan Tian, Yiran Dong, Xuelian Sun, Hufeng Xu, Dong Zhang

**Affiliations:** 1 Experimental and Translational Research Center, Beijing Friendship Hospital, Capital Medical University, Beijing, 100050 China; 2 Beijing Clinical Research Institute, Beijing, 100050 China; 3 Beijing Key Laboratory of Tolerance Induction and Organ Protection in Transplantation, Beijing, 100050 China; 4 National Clinical Research Center for Digestive Diseases, Beijing, 100050 China; 5 Department of Emergency Medicine, Beijing Friendship Hospital, Capital Medical University, Beijing, 100050 China

## Abstract

CD4^+^ T-cell-converted CD4^−^CD8^−^ double negative (cDNT) have strong suppressive activity in the maintenance of immune tolerance, whereas IL-2 promotes cDNT proliferation and enhances cDNT resistance to apoptosis. However, the intrinsic mechanisms that regulate the survival of cDNT are still unknown. Here we demonstrate that the *OX40* molecule was highly expressed on cDNT. The expression of *OX40* was necessary to promote proliferation and inhibit apoptosis of cDNT in vivo and in vitro. *OX40* promoted the survival of cDNT by regulating the expression of Bcl-2, Bcl-xL, Survivin, and BCL2L11. Canonical NF-κB cell signaling played an important role in the transmission of essential division and survival signals through *OX40* in cDNT. IL-2 promoted the survival of cDNT in part via elevating the expression of the *OX40* molecule. IL-2 promoted *OX40* expression via downregulating the PPARα expression. In conclusion, we elucidated that *OX40* is a key molecule that regulates cDNT proliferation and survival. IL-2 promoted *OX40* expression by downregulating the PPARα binding to the *OX40* promoter, leading to the elevated expression of Bcl-2, Bcl-xL, and Survivin in cDNT, which finally resulted in the promoted proliferation and decreased apoptosis of cDNT.

## Introduction

Regulatory CD4^−^CD8^−^ double-negative T cells (DNT), which express αβ T-cell receptor (TCR) but do not express natural killer (NK) cell markers compose only a small population of T lymphocytes (1–5%) in the peripheral blood and lymphoid organs of rodents and humans^1,2^. DNT cells have strong suppressive activity toward CD4^+^ T cells and CD8^+^ T cells^3–6^, as well as B cells^4,7^, dendritic cells (DCs)^8^, and NK cells^9^, which are capable of suppressing the immune response and exert significant protection against allograft rejection, graft-versus-host disease, and autoimmune diseases^3,6,10–13^.

We have identified the differentiation pathway from CD4^+^ T cells to DNT, which are important for maintaining immune system homeostasis^3,14^. The DNT can be derived from activated and proliferated CD4^+^ T cells, which stimulated by bone marrow-derived DCs in vitro^4^. The over-activated CD4^+^ T cells can also be converted into DNT in vivo^15^. The CD4 T-cell-converted DNT (cDNT) are CD25^+^, CD44^+^, CD69^+^, and *Foxp3*^−^. These cDNT potently suppressed vigorous allo- and autoimmune responses, prolonged islet and skin allograft survival, and prevented and cured autoimmune type 1 diabetes with antigen-specificity^3,6,16^.

Interleukin (IL)-2 is a critical regulator of the activation and proliferation of T lymphocytes, also plays an important role in the generation and expansion of cDNT^3^. cDNT cells were in an anergic state, whereas exogenous IL-2 could restore cDNT responses, promote cDNT proliferation, and enhance cDNT resistance to Activation Induced Cell Death (AICD)^17^. However, the intrinsic mechanism that regulates the survival of cDNT and how IL-2 promotes cDNT proliferation and enhance cDNT resistance to AICD remain unknown.

*OX40*, often recognized as a costimulatory receptor for T cells, is predominantly expressed on activated CD4 T cells. It is essential for regulating the conventional CD4 and CD8 T-cell division, differentiation, and survival. Previous studies reported that the cytokines IL-1, IL-2, IL-4, and tumor necrosis factor stimulation could enhance or prolong *OX40* expression^15,18^. *OX40* is also found on *Foxp3*^+^ Tregs, but it is dispensable for the genesis and suppressor functions of naturally arising CD4^+^*Foxp3*^+^ Tregs^19^. However, the expression and function of *OX40* in DNT are still unknown. In this study, we have identified *OX40* as the key regulator of cDNT survival and the mediator of IL-2 on the promotion of proliferation and resistance to AICD of cDNT.

## Results

### *OX40* molecule was highly expressed on cDNT and was necessary to promote proliferation and inhibit apoptosis of cDNT

**Fig. 1:**
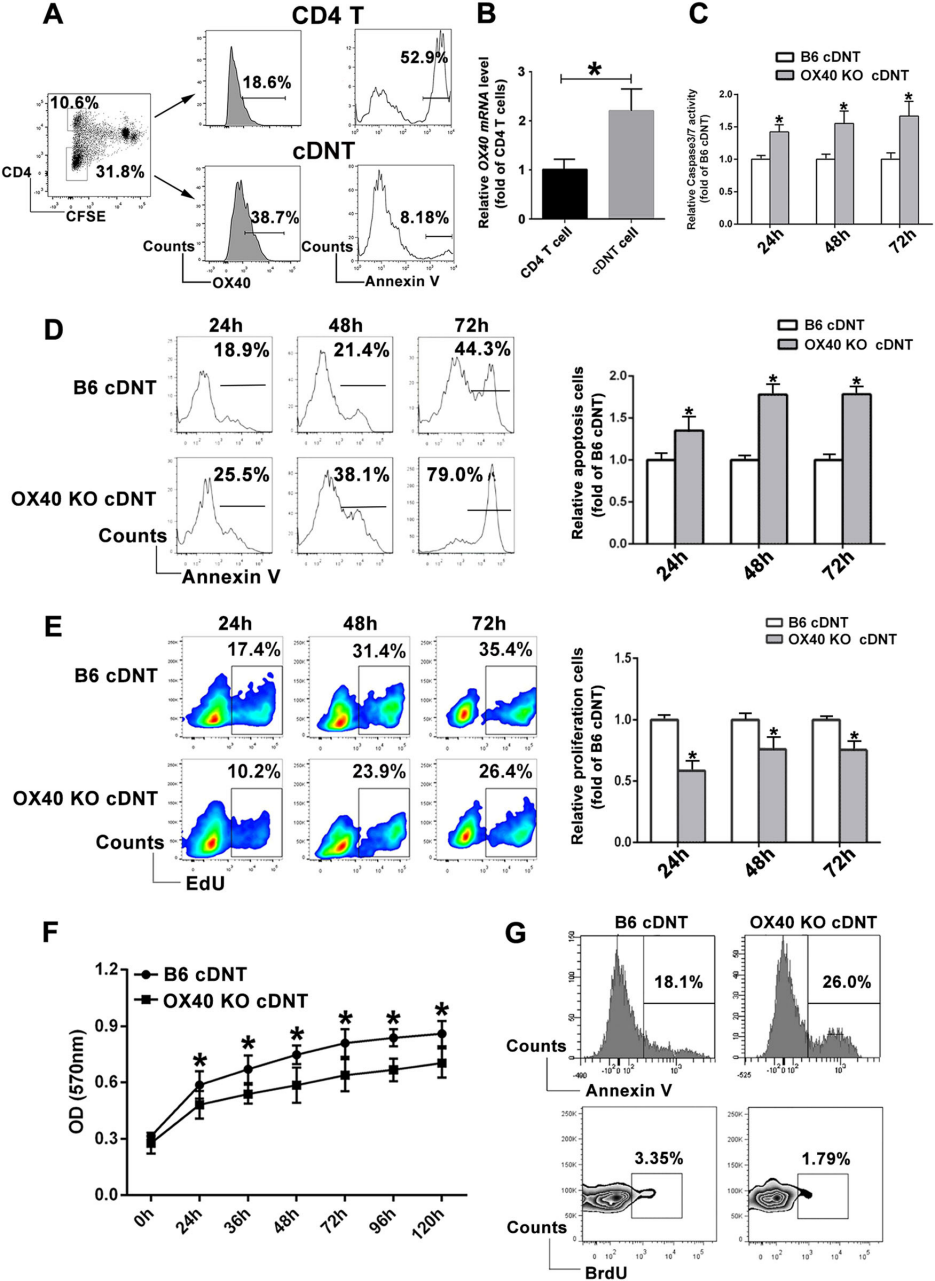
*OX40* regulated survival of cDNT. **a** CD4^+^CD25^−^ T cells from C57BL/6 mice were stimulated with mature DBA/2 DCs for 7 days. The converted DNT and activated CD4^+^ T cells were detected for *OX40* expression through flow cytometric analysis. Annexin V staining was used to detect apoptosis of the two cell populations. **b** The relative mRNA expression of *OX40* was determined by real-time PCR in activated CD4^+^ T cells and cDNT. **c** Caspase 3/7 activation was determined in B6 cDNT or *OX40* KO cDNT after being stimulated with anti-CD3/CD28 antibodies for 24, 48, and 72 h. **d** The converted C57BL/6 and *OX40* KO DNT were incubated with anti-CD3/CD28 antibodies for 24, 48, and 72 h, and apoptosis was assessed via Annexin V staining. A representative flow cytometry image of Annexin V^+^ cells (% cDNT) from each group is shown (left). Statistical analysis of Annexin V^+^ cells in *OX40* KO cDNT relative to B6 cDNT in each group was determined by flow cytometry (right). **e** The converted B6 and *OX40* KO cDNT were incubated with anti-CD3/CD28 antibodies for 24, 48, and 72 h, and proliferation was assessed via EdU incorporation. Representative flow cytometry image of EdU^+^ cells (% cDNT) from each group is shown (left). In addition, statistical analysis was determined by flow cytometry (right). **f** The B6 cDNT and *OX40* KO cDNT stimulated with anti-CD3/CD28 antibodies were incubated with Alamar Blue, and the absorbance at 570 nm at different time points was measured. **g** A total of 5 × 10^6^ converted DNT or *OX40* KO DNT were adoptively transferred into B6D2F1 (*H-2*^*b/d*^) recipient mice by tail vein injection. Three days after injection of BrdU (100 µg/day), splenocytes were isolated, and stained with fluorochrome-conjugated antibodies against mouse H2D^d^ and CD3, and the cDNT (H2D^d−^CD3^+^) apoptosis and proliferation were determined by Annexin V and BrdU staining in vivo. Data are representative of three experiments with similar results. The data are depicted as the mean ± SD, *n* = 5 in each group. *p < 0.05

As we reported^3^, after 7 days’ in vitro stimulation with mature DCs, approximately 30% of CD4 T cells lost CD4 expression and became DNT (Fig. 1a, left). By monitoring the apoptosis of activated CD4^+^ and cDNT, we found that the percentage of Annexin V^+^ cells was markedly lower in the cDNT than in activated CD4^+^ T cells (51.7 ± 5.7% vs. 8.1 ± 4.2%, *P* < 0.05, Fig. 1a, right). Meanwhile, *OX40* expression was significantly higher in cDNT than that in activated CD4^+^ T cells (37.3 ± 5.91% vs. 18.9 ± 4.59%, *P* < 0.05; Fig. 1a, middle). Furthermore, cDNT and CD4^+^ T cells were sorted from mixed lymphocyte reaction (MLR) and were assessed for *OX40* mRNA expression. As shown in Fig. 1b, the *OX40* mRNA expression level of cDNT was also significantly higher than that of CD4^+^ T cells. No significant differences of CD27, CD28, CD30, CD40, CD95, and ICOS expression between CD4^+^ T cells and cDNT (supplementary Figure 1), indicating that *OX40*, the apoptosis-related gene of activated CD4^+^ and CD8^+^ T cells, may also play an important role on the survival of cDNT.

To further investigate the functional potential of *OX40* on the converted DNT survival, *OX40* knockout (KO) DNT converted from *OX40* KO CD4^+^ T cells were stimulated with anti-CD3 and CD28 antibodies in vitro, and the apoptotic and proliferation rate was analyzed with Annexin V and EdU incorporation on 24, 48, and 72 h, respectively. Compared with B6 cDNT, caspase 3/7 activity in *OX40* KO cDNT was increased (Fig. 1c), and the apoptotic rates of *OX40* KO cDNT were significantly higher (Fig. 1d). In contrast, proliferation was decreased in *OX40* KO cDNT determined by EdU incorporation (Fig. 1e). We also measured the proliferation of cDNT using an Alamar Blue cell viability reagent, and as shown in Fig. 1f, C57BL/6 (B6) cDNT had higher fluorescent, which indicated that cDNT had a higher proliferation rate than *OX40* KO cDNT.

In addition, we also evaluated the regulation of *OX40* on the proliferation and apoptosis of converted DNT in vivo. A total of 5 × 10^6^ converted wild-type (WT) or *OX40* KO DNT were adoptively transferred into B6D2F1 (*H-2*^*b/d*^) recipient mice by tail vein injection. After 3 days injection of BrdU (100 µg/day/mouse), splenocytes were isolated and stained with fluorochrome-conjugated antibodies against mouse H2D^d^, the proliferation of cDNT (H2D^d^-negative cells) was at a lower level when *OX40* was deficient; however, the apoptosis of *OX40*-deficient cDNT was increased (Fig. 1g).

The collective data from in vitro and in vivo studies indicated that the expression of *OX40* was necessary to promote proliferation and inhibit apoptosis of cDNT.

### *OX40* promoted the survival of cDNT by regulating the expression of Bcl-2, Bcl-xL, Survivin, and Bcl-2-like protein 11

**Fig. 2:**
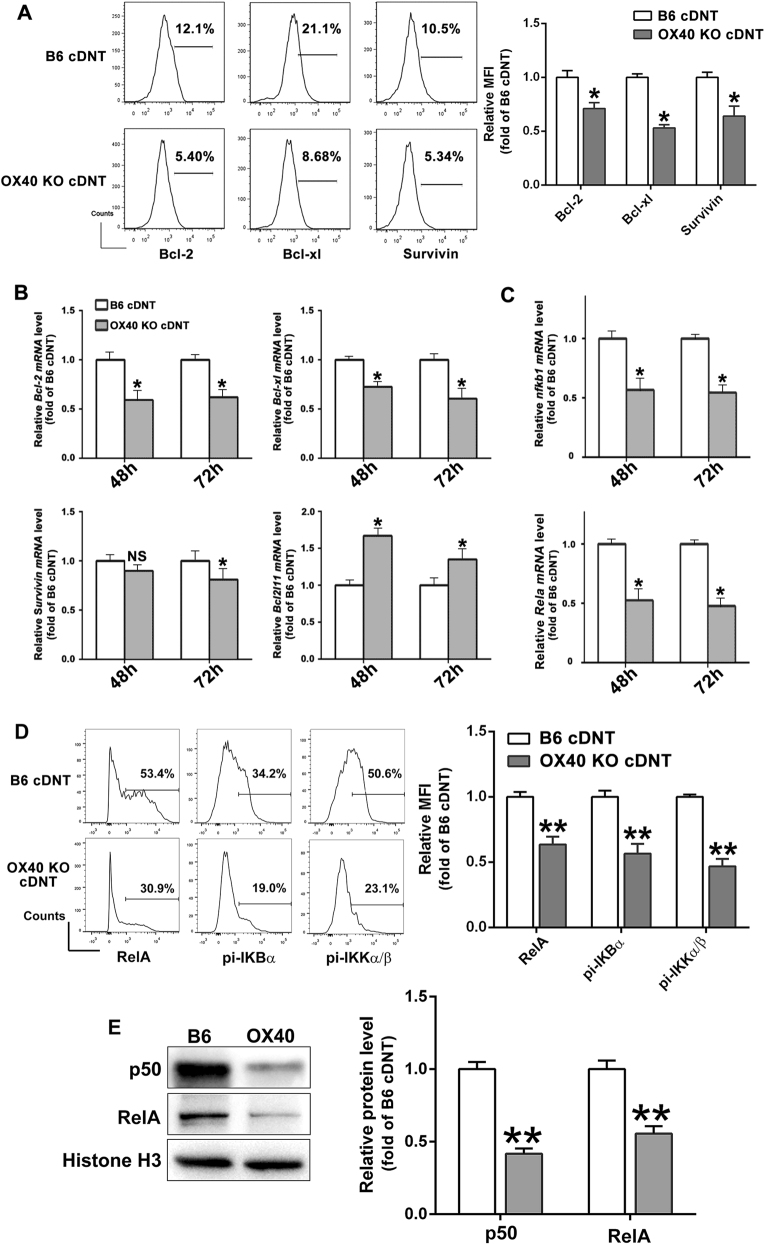
*OX40* controls cDNT survival by its regulation on Survivin, Bcl-2, Bcl-xL, and BCL2L11. **a** Flow cytometric analysis of Bcl-2, Bcl-xl, and Survivin protein expression in B6 cDNT and *OX40* KO cDNT after anti-CD3/CD28 stimulation for 48 h. **b** Relative mRNA expression of anti-apoptosis genes (*Bcl-2*, *Bcl-xl*, and *Survivin*) and pro-apoptosis genes (*Bcl2l11*) in B6 cDNT and *OX40* KO cDNT after incubation with CD3/CD28 for 48 and 72 h. **c** The relative mRNA levels of NF-κB signaling genes (*nfkb1* and *rela*) in *OX40* KO cDNT compared with WT cDNT. **d** Flow cytometric analysis of RelA, and phosphorylation of IκBα and IKKα/β protein expression in B6 cDNT and *OX40* KO cDNT cells after anti-CD3/CD28 stimulation for 48 h. **e** Nuclear protein extracts were prepared from B6 cDNT cells and *OX40* KO cDNT cells after anti-CD3/CD28 stimulation for 48 h. The RelA and p50 protein levels in the cells were analyzed by western blot. The relative density of the RelA and p50 band was normalized to Histone H3. The data are presented as the mean ± SD, *n* = 5 in each group. *p < 0.05. NS no significance

*OX40* engagement promotes CD4^+^ T-cell survival through the induction of the anti-apoptotic molecules, Bcl-2, Bcl-xL^20^, and cell cycle progression-related protein Survivin^21^. Meanwhile, it also greatly enhances CD8^+^ T-cell survival by upregulating Bcl-xL^22^. Ligation of *OX40* on neutrophils results in enhanced survival, which correlates with reduced activation of caspase 3 and with augmented levels of anti-apoptotic markers^23^. To garner further insight into the molecular mechanism by which *OX40* modulated cDNT survival, the apoptosis-related proteins Bcl-2, Bcl-xL, Survivin, and Bcl-2-like protein 11 (BCL2L11) were analyzed on converted WT DNT and *OX40* KO DNT. cDNT were cultured up to 72 h with anti-CD3 and CD28 antibodies in vitro, the apoptosis of *OX40* KO cDNT was increased, together with significant reduction of anti-apoptotic molecules, Bcl-2, Bcl-xL, and Survivin (Fig. 2a) when *OX40* was knocked out. In addition, as shown in Fig. 2b, Bcl-2, Bcl-xL, and Survivin mRNA expression were dramatically reduced in *OX40* KO cDNT, whereas BCL2L11 mRNA expression was augmented. These data indicated that *OX40* could promote cDNT survival through the induction of apoptosis-related protein Bcl-2, Bcl-xL, and Survivin, and the suppression of pro-apoptotic protein BCL2L11.

Canonical nuclear factor (NF)-κB cell signaling plays an important role in the transmission of essential division and survival signals through *OX40* in T cells^24,25^. Therefore, we examined the NF-κB1 and RelA mRNA expression of cDNT after 48 and 72 h cultures in vitro. The mRNA expression of NF-κB1 and RelA of cDNT were significantly reduced when *OX40* was deficient (Fig. 2c). We also detected NF-κB signaling pathway through flow cytometry and western blotting at protein level. As shown in Fig. 2d, *OX40* deficiency could downregulate phosphorylation events of IKKα/β, IκBα, and RelA (p65) expression of cDNT. Meanwhile, western blotting results also showed *OX40* KO cDNT decreased p50 and RelA expression in the nucleus (Fig. 2e), suggesting that canonical NF-κB signaling was also important for transmission of proliferation and survival signals through *OX40* in cDNT.

### IL-2 promoted the survival of cDNT in part via elevating the expression of *OX40* molecule

**Fig. 3:**
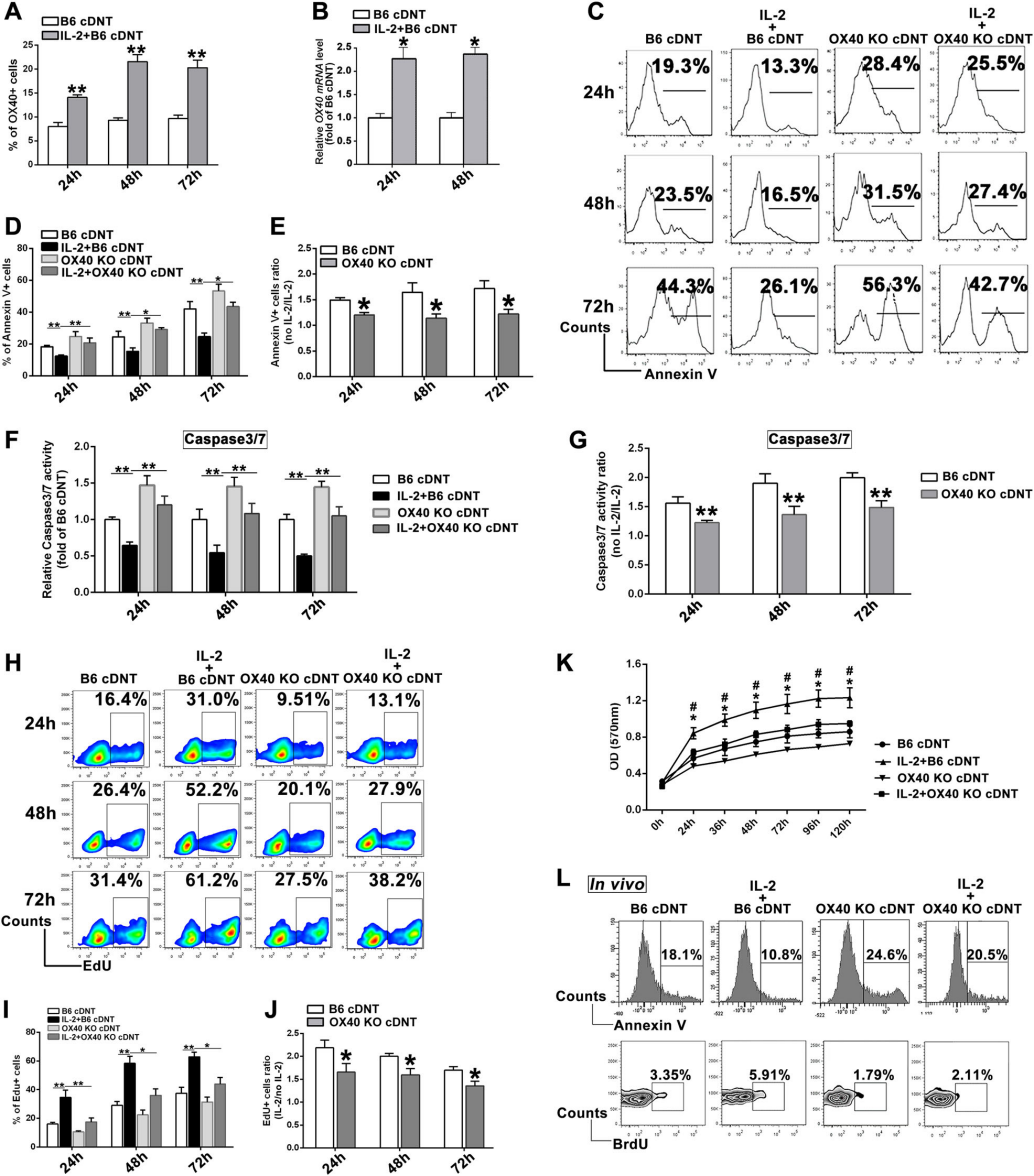
IL-2 promoted cDNT survival through *OX40* molecule. **a** Statistical analysis of *OX40* expression on cDNT with or without IL-2 for 24, 48, and 72 h based on flow cytometric analysis. **b** Relative changes in *OX40* expression induced by IL-2 in cDNT were plotted as fold changes of B6 cDNT without IL-2 stimulation. **c** B6 cDNT and *OX40* KO cDNT were stimulated with or without IL-2 for 24, 48, and 72 h, and apoptosis of the cells was detected through Annexin V staining. Representative flow cytometric image of Annexin V^+^ cDNT from each group. **d** Statistical analysis of Annexin V^+^ cells in each group at a different time point. **e** Relative changes of Annexin V^+^ cells with or without IL-2 in B6 cDNT were compared with that in *OX40* KO cDNT at 24, 48, and 72 h. **f** Caspase 3/7 activation was determined in B6 cDNT and *OX40* KO cDNT after stimulation with or without IL-2. **g** The relative changes of Caspase 3/7 activation with or without IL-2 in B6 cDNT were compared with the ratio in *OX40* KO cDNT at 24, 48, and 72 h. **h** The percentages of EdU^+^ cells relative to the total numbers of B6 cDNT or *OX40* KO cDNT with or without IL-2 was determined by flow cytometry in the indicated groups. **i** Statistical analysis of EdU^+^ cells in each group at different time points. **j** Relative changes of EdU^+^ cells with or without IL-2 in B6 cDNT were compared with that in *OX40* KO cDNT at 24, 48, and 72 h. **k** The B6 cDNT and *OX40* KO cDNT were stimulated with IL-2 or without IL-2, and Alamar Blue was incubated during the stimulation. The proliferations of the cells were detected at the absorbance of 570 nm at different time points. ^#^*P* < 0.05, IL-2 + B6 cDNT versus B6 cDNT; ^*^P < 0.05, IL-2 + B6 cDNT versus IL-2 + *OX40* KO cDNT. **l** A total of 5 × 10^6^ B6 cDNT or *OX40* KO cDNT were adoptively transferred into B6D2F1 recipient mice by tail vein injection in the presence or absence of IL-2/Fc protein. After 3 days, cDNT were analyzed by flow cytometry for proliferation and apoptosis with BrdU and Annexin V detection. Data are representative of three experiments with similar results. The data are presented as the mean ± SD, *n* = 5 in each group. *p < 0.05, **p < 0.01, NS no significance

IL-2 is critical for the activation and proliferation of T cells, plays an important role in the generation and expansion of cDNT. In this study, we found that IL-2 could remarkably promote the expression of *OX40* in cDNT (Fig. 3a, b), however, CD27, CD28, CD30, CD40, CD95, and ICOS were not influenced by IL-2 (supplementary Figure 2). Therefore, we tested whether the regulation of IL-2 on cDNT proliferation and apoptosis was *OX40*-dependent. Accordingly, converted WT DNT and *OX40* KO DNT were stimulated with anti-CD3 and CD28 antibodies in vitro with or without rIL-2. With exogenous IL-2, the apoptosis rates of cDNT from WT B6 mice and *OX40* KO mice were both decreased (Fig. 3c, d); however, the reduction of apoptosis in converted WT B6 cDNT was markedly greater than that in *OX40* KO cDNT (Fig. 3e). IL-2 could inhibit the activity of caspase 3/7 in cDNT; however, the reduction of caspase 3/7 activity of cDNT with rIL-2 stimulation in vitro was impaired when *OX40* was deficient (Fig. 3f, g). Similarly, the proliferation of cDNT was significantly augmented in the presence of rIL-2 stimulation (Fig. 3h, i). Nevertheless, the proliferation enhancements of cDNT with rIL-2 stimulation in vitro were reduced when *OX40* was knocked out (Fig. 3j). The Alamar Blue cell proliferation assay further supported that *OX40* was involved in the regulation of IL-2 on the survival of cDNT (Fig. 3k).

Additionally, B6D2F1 mice underwent adoptive transfer with 5 × 10^6^ B6 cDNT or *OX40* KO cDNT in the presence or absence of IL-2/Fc. Three days after injection of BrdU, cDNT were analyzed by flow cytometry for proliferation and apoptosis with BrdU and Annexin V detection. As shown in Fig. 3l, the effect of *OX40* on IL-2-regulating cDNT survival in vivo was comparable to those in vitro.

**Fig. 4:**
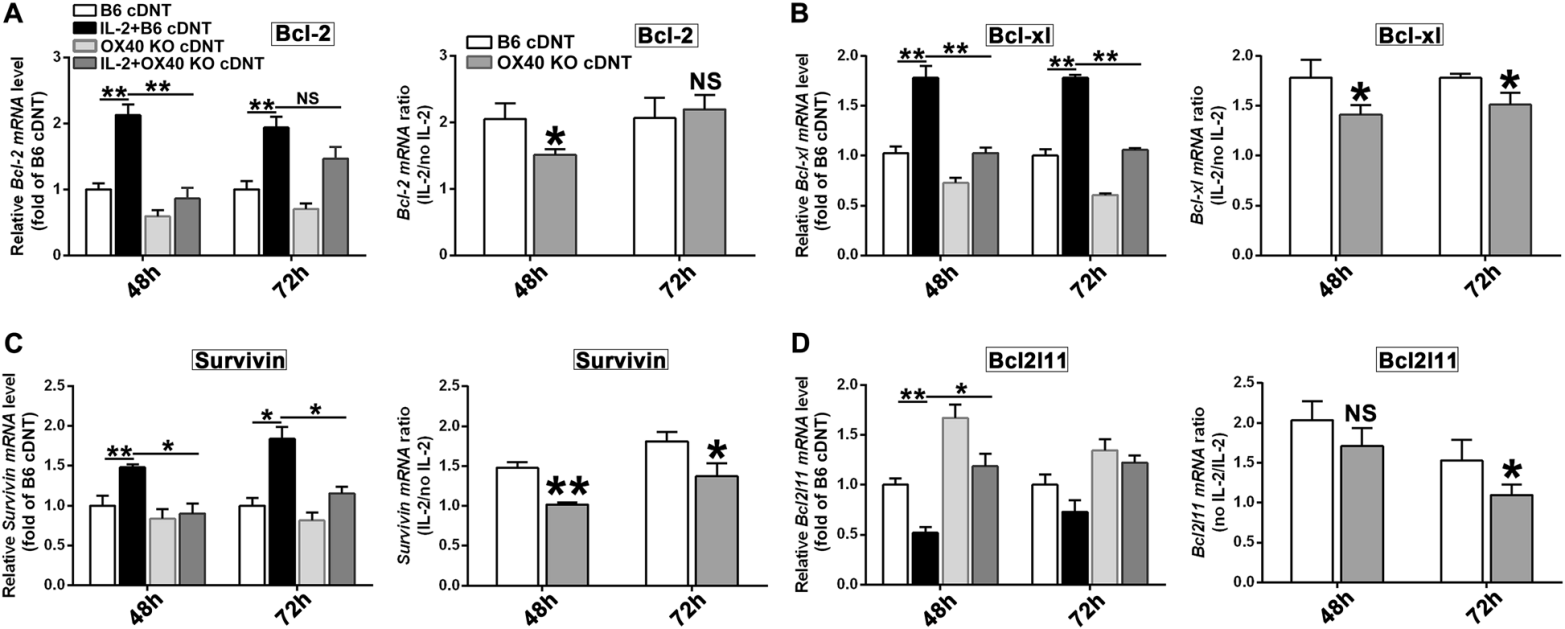
Apoptosis-related genes were affected in *OX40*-deficient cDNT under IL-2 stimulation. **a** Relative mRNA expression of anti-apoptosis genes *Bcl-2* (**a**), *Bcl-xl* (**b**), and *Survivin* (**c**), and pro-apoptosis genes *Bcl2l11* (**d**) in B6 cDNT and *OX40* KO cDNT stimulated with IL-2 or without IL-2 at different time points. Relative changes of the genes in the presence or absence of IL-2 in B6 cDNT compared with that in *OX40* KO cDNT. The data are presented as the mean ± SD, *n* = 5 in each group. *p < 0.05, **p < 0.01, NS no significance

We also noticed that rIL-2 upregulated anti-apoptotic molecules, *Bcl-2* (Fig. 4a), *Bcl-xl* (Fig. 4b), and *Survivin* (Fig. 4c) expression and downregulated pro-apoptotic gene *Bcl2l11* expression (Fig. 4d) of cDNT. However, the regulation of IL-2 on these apoptotic-related genes was impaired in *OX40*-deficient cDNT (Fig. 4a–d). These data suggested that IL-2 promoted the survival of cDNT at least in part via the elevation of *OX40* molecule expression.

### IL-2 promoted *OX40* expression by downregulating the PPARα expression

**Fig. 5:**
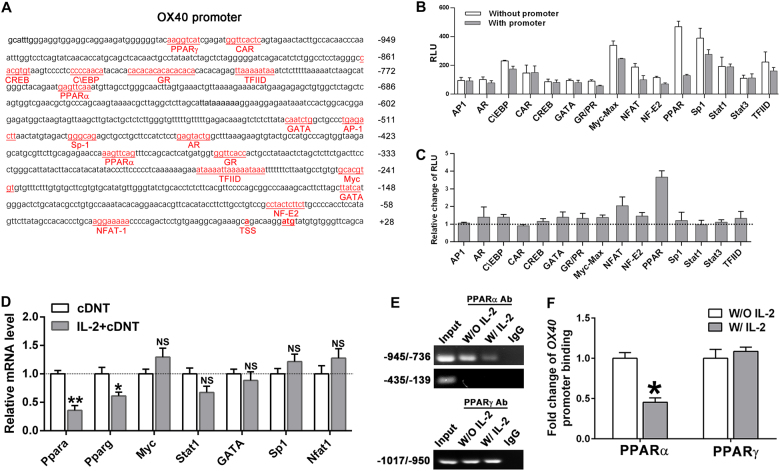
Identification of the core promoter of mice *OX40* gene. **a** Nucleotide sequence of the *OX40* promoter region. Potential regulatory elements identified according to the Transcription Factor Binding Sites database TRANSFAC are shown underlined and identified by the appropriate symbols. **b** Promoter-binding transcription factor (TF) profiling array assay of mice *OX40* core promoter was performed. This is a competitive binding assay performed to identify promoter-bound TFs through comparisons of the results in the presence (with promoter) or absence (without promoter) of the mouse *OX40* core promoter. If the *OX40* promoter contains a TF-binding sequence, it will display a lower chemiluminescence activity. **c** Relative change of chemiluminescence activity in the absence (control) of the *OX40* core promoter with presence (control + promoter) of the promoter. **d** Relative mRNA levels of the transcription factors in the presence or absence of IL-2. The data are presented as the mean ± SD, *n* = 3 in each group. *p < 0.05, **p < 0.01, NS no significance. **e** ChIP analysis of cDNT stimulated with IL-2 (50 ng/ml) for 48 h. Conventional PCR was performed to measure the relative levels of the antibody-bound DNA fragments. Soluble chromatin from the cDNT was immunoprecipitated with the PPARα, PPARγ antibody, or incubated with normal rabbit serum (IgG) for control purposes. The amplification for soluble chromatin before immunoprecipitation are shown as input. **f** Real-time PCR was used to determine the levels of PPARα and PPARγ bound directly to *OX40* promoter sequence (−945/−736 bp and −1017/−950 bp upstream from transcription start site). The data are presented as the mean ± SD, *n* = 5 in each group. *p < 0.05, NS no significance

To investigate the intrinsic mechanism of IL-2 regulation on *OX40* molecular expression in cDNT, the upstream factors regulating *OX40* were investigated. In the *OX40* promoter region (−1032/+28 region), potential transcription factor (TF)-binding sites are presented in Fig. 5a and were based on bioinformatic prediction. To further identify the TFs that bind to the *OX40* core promoter region and regulate the activation of the *OX40* gene, a competitive promoter-binding TF profiling array for the *OX40* core promoter in cDNT was performed. Among the potential TF-binding sites, PPAR has relative higher changes of chemiluminescence activity between the absence (control) of *OX40* core promoter vs. presence (control + promoter) of the promoter within the *OX40* promoter (Fig. 5b, c). Furthermore, PPARα and PPARγ mRNA expression was markedly decreased in the cDNT under IL-2 stimulation (Fig. 5d).

Then we performed ChIP assays with the *OX40* promoter to test whether PPARα or PPARγ was associated with the *OX40* gene upregulation of cDNT in the presence of IL-2. As shown in Fig. 5e, after immunoprecipitation with PPARα antibodies, a positive band of approximately −945/−736 bp was yielded in the bound DNA of cDNT without IL-2 stimulation, however, lower amplification of bound DNA from cDNT with IL-2 stimulation was shown. Precipitation with PPARα or without antibody did not show significant changes of positive DNA band (approximately −435/−139 bp). Precipitation with PPARγ antibody or without antibody did not show significant changes of positive DNA band between cDNT treated with or without IL-2, which strongly suggested that PPARα is able to bind to the *OX40* promoter and is involved in regulation of *OX40* expression by IL-2. Real-time PCR further proved that PPARα direct binding to *OX40* promoter sequence (−945/−736 bp) was reduced in cDNT treated with IL-2 compared to cDNT without IL-2 stimulation.

**Fig. 6:**
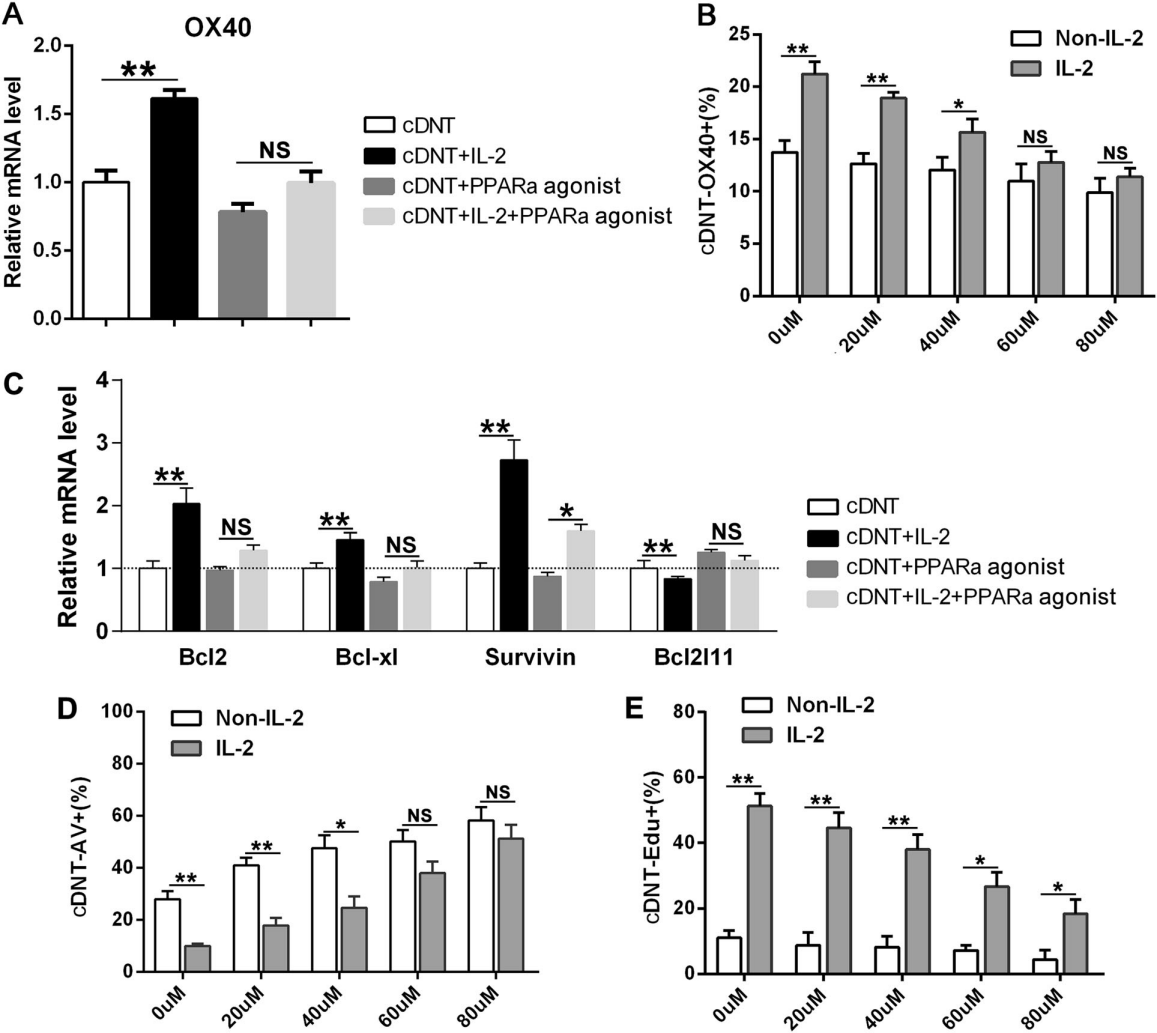
IL-2 regulated *OX40* expression through transcription factor PPARα. **a** Inhibition of *OX40* mRNA levels after incubation with or without IL-2 for 48 h by a PPARα agonist (80 µM). **b** The percentage of *OX40*^+^ cDNT was detected after stimulation with a PPARα agonist in the presence or absence of IL-2. Statistical analysis of *OX40*^+^ cells proportion stimulation with IL-2 relative to the proportion without IL-2 in each group, as determined by flow cytometry. **c** Relative mRNA levels of anti-apoptosis genes (*Bcl-2*, *Bcl-xl*, and *Survivin*) and pro-apoptosis genes (*Bcl2l11*) of cDNT after incubation with a PPARα agonist with or without IL-2. Statistical analysis of the genes expression stimulation with IL-2 relative to the genes without IL-2 in each group was determined. **d** Apoptosis of cDNT (Annexin V^+^) was detected after stimulation with a PPARα agonist with or without IL-2 by flow cytometry. **e** The proliferation of cDNT (EdU^+^) was detected after stimulation with a PPARα agonist with or without IL-2 by flow cytometry. The data are presented as the mean ± SD, *n* = 5 in each group. *p < 0.05, **p < 0.01, NS no significance

To ascertain the possible involvement of PPARα activity in regulation of IL-2 on *OX40* molecule, the cultures were performed in the presence or absence of the PPARα agonist and rIL-2. As an aforementioned result, *OX40* expression and proliferation of cDNT were significantly augmented in the presence of rIL-2 stimulation; however, the upregulation of *OX40* expression induced by IL-2 was abrogated by a PPARα agonist dose dependently (Fig. 6a, b). In addition, the effect of rIL-2 on the upregulation of anti-apoptotic molecules, Bcl-2, Bcl-xl, and Survivin, or the downregulation of the pro-apoptotic gene *Bcl2l11* were all impaired in the presence of a PPARα agonist (Fig. 6c). Unquestionably, PPARα agonist neutralized the anti-apoptotic effect of IL-2 on cDNT, the percent of apoptotic rate was increased in the presence of a PPARα agonist (Fig. 6d). In contrast, the proliferation rates of cDNT with rIL-2 stimulation in vitro were reduced in the presence of a PPARα agonist, and the changes were dose-dependent (Fig. 6e).

Taken together, these results suggested that IL-2 promoted *OX40* expression by downregulating the PPARα expression, leading to the elevated expression of Bcl-2, Bcl-xL, and Survivin in cDNT, which finally resulted in promoted proliferation and decreased apoptosis.

## Discussion

*OX40* is induced primarily during T-cell activation and plays an important role in the survival and proliferation of T cells^26,27^. *OX40* is mainly expressed on activated T cells and is preferentially expressed on CD4^+^ T cells, although activated CD8^+^ T cells also express *OX40*, albeit at lower levels^24^. *OX40* is also highly expressed on both natural and induced *Foxp3*^+^ Tregs; however, in contrast to its costimulatory role to T effector cells, *OX40* is a rather potent negative regulator of *Foxp3*^+^ Tregs^25,28^. *OX40* costimulation did not influenced the survival of the *Foxp3*^+^ Tregs significantly, but markedly inhibited *Foxp3* gene expression^25^.

In this study, we have demonstrated for the first time that *OX40* is highly expressed on cDNT; moreover, similar to its costimulatory role to activated T effector cells, unlike *Foxp3*^+^ Tregs, *OX40* is also a potent regulator on cDNT proliferation and survival. *OX40*-deficient cDNT had a higher apoptotic rate and lower proliferation rate than WT cDNT. *OX40* participated into cDNT survival regulation by inducting the expression of apoptotic-related proteins Bcl-2, Bcl-xL, and Survivin, thus suppressing the expression of pro-apoptotic gene *BCL2L11*.

*OX40* signaling can target the canonical NF-κB (NF-κB1) pathway in peripheral antigen-responding CD4 T cells^24,25^. In this study, we also identified significantly lower phosphorylation events of IKKα/β, IκBα, RelA (p65) expression, and p50, RelA nuclear import in *OX40*-deficient cDNT accompanied by the changes of Bcl-2 and Bcl-xL indicating that *OX40* might promote the survival of cDNT by regulating the expression of Bcl-2 and Bcl-xL via NF-κB cell signaling. *OX40* signals also controls Survivin expression of cDNT. Survivin, a member of the inhibitor of apoptosis family, was found to bind the active forms of the executioner proteases caspase 3 and 7, but not the upstream initiator caspase 8, and this inhibited apoptosis induced by overexpression of procaspase 3 and 7. Moreover, cotransfection of Survivin prevented spontaneous processing of caspase 7 and caspase 3 to their active forms^29^. In CD4^+^ T cells, blocking Survivin suppressed S-phase transition and division of T cells and led to apoptosis^21^. Converted DNT highly that expressed Survivin may also have contributed to cDNT proliferation and anti-apoptosis.

Research over the past decade has definitively shown the importance of *OX40*–*OX40*L interactions in development of immune-mediated disease^24^, based on that the *OX40*–*OX40*L checkpoint inhibition may have potential clinical application in the treatment in autoimmune diseases. However, because anti-*OX40*/*OX40*L has complicated effects on different cell types, the use of *OX40*–*OX40*L checkpoint inhibition should be cautious, especially in the combination with cDNT-based cell therapy.

IL-2 plays an unequivocal role in priming activated T cells to undergo apoptotic cell death, which serves as a critical feedback regulator of clonal expansion^30^; however, accumulating evidence suggests that IL-2 is essential for the activation and suppressive function of *Foxp3*^+^ Tregs^31–33^ and is sufficient to induce proliferation of *Foxp3*^+^ Tregs. Converted DNT were hypo-responsive when stimulated by mature DCs, and IL-2 promoted the expression of *OX40* in cDNT. Meanwhile, IL-2 completely restored their responsiveness, and enhanced cDNT proliferation. In addition, IL-2 increased the resistance of cDNT to apoptosis. However, the regulation of IL-2 on cDNT proliferation and apoptosis was impaired when *OX40* in cDNT was knocked out. Furthermore, the mRNA expression of Bcl-2, Bcl-xL, and Survivin was reduced in *OX40*-deficient cDNT, even in the presence of IL-2, whereas mRNA expression of BCL2L11 was augmented. These data suggested that IL-2 promoted the survival of cDNT in part via elevation of the expression of the *OX40* molecule.

The regulation of IL-2 on *OX40* upregulation of cDNT is unknown. In this study, we provide evidence that this regulation is PPARα-dependent. Promoter-binding TF profiling arrays revealed that a possible presence of binding site for PPARα and PPARγ within the *OX40* promoter. The ChIP assay further supported that PPARα but not PPARγ is able to bind to the *OX40* promoter and involves in the negative regulation of *OX40* expression in cDNT. Furthermore, *OX40* expression of cDNT were significantly augmented in the presence of rIL-2 stimulation. In contrast, the *OX40* expression of cDNT with rIL-2 stimulation in vitro was reduced when a PPARα agonist was presented.

The PPARs are members of the nuclear-hormone-receptor superfamily; they transduce a wide variety of signals, including environmental, nutritional, and inflammatory events. Genetic ablation of PPARα or PPARγ on CD4 T cells results in higher production of interferon γ and IL-2 through PPAR-mediated transrepression activities^34^. In this study, we found that the administration of IL-2 effectively promoted *OX40* expression in cDNT through a PPARα-dependent process and consequently enhanced Bcl-2, Bcl-xL, and Survivin expression, and finally enhanced survival of cDNT. However, how IL-2 affects PPARα activities needs to be further studied.

In conclusion, we elucidated that *OX40* is a key molecule that controls CD4 T-cell-converted DNT proliferation and apoptosis by upregulation of Survivin, Bcl-2, and Bcl-xL, and downregulation of pro-apoptotic gene *BCL2L11*. IL-2 promotes cDNT proliferation and resistance to apoptosis in part by upregulation of *OX40*. This regulation of IL-2 on *OX40* expression in cDNT is controlled by PPARα. These Snew findings may have important implications in cDNT-based cell therapy for the future.

## Materials and Methods

### Mice

Male C57BL/6 (*H-2*^*b*^) mice, C57BL/6 *OX40* KO (*H-2*^*b*^) mice, DBA/2 (*H-2*^*d*^) mice, and B6D2F1 (*H-2*^*b/d*^) mice were obtained from the Jackson Laboratory (Bar Harbor, ME, USA). The mice were maintained in a pathogen-free, temperature-controlled environment under a 12-h light/dark cycle at the Beijing Friendship Hospital, and all animal protocols were approved by the Institutional Animal Care and Ethics Committee.

### Antibodies and reagents

Recombinant mouse IL-2 (rmIL-2) and granulocyte-macrophage colony-stimulating factor were purchased from PeproTech (Rocky Hill, NJ, USA). Mouse IL-2/Fc fusion protein (a long-lasting form of IL-2) was obtained from Jiangsu Futai Biotechnology (Jiangsu, China). Fluorochrome-conjugated antibodies against mouse CD3, CD4, CD8, NK1.1, TCRγδ, CD86, *OX40*, Bcl-2, H2D^d^, isotype controls, and BrdU staining kits were obtained from eBioscience (San Diego, CA, USA). Anti-mouse PE-Bcl-xL and Survivin were obtained from CST (Boston, MA, USA). Anti-mouse p105/p50, p65, and IKB alpha (phosphor S36) were obtained from Abcam (Cambridge, MA, USA), while anti-mouse phosphor-IKKα/β (Ser176/180) and Histone H3 were obtained from CST. Purified anti-CD3, CD28 antibodies, and Annexin V-PE antibodies were purchased from BD Pharmingen (San Diego, CA, USA). Paraformaldehyde, saponin, Triton X-100, and PPARα agonist (WY-14643) were obtained from Sigma (St. Louis, MO, USA). A mouse T-cell enrichment column was purchased from R&D Systems (Minneapolis, MN). Anti-PE microbeads, and magnetic bead separation columns were obtained from Miltenyi Biotec (Auburn, CA). The ChIP assay kit was from Millipore (Bedford, MA, USA).

### Conversion of DNT in vitro

The generation of CD4 T cells converted to DNT was described previously^3^. Briefly, CD4^+^CD25^−^ T cells were isolated from spleens and lymph nodes of C57BL/6 mice or C57BL/6 *OX40* KO mice. The purified CD4^+^CD25^−^ T cells were then cultured with DBA/2 mature DCs and rmIL-2 (50 ng/ml) for 7 days. Converted DNT were sorted from culture through a fluorescence-activated cell sorter (FACS Aria II; BD Biosciences, San Diego, CA, USA).

### Re-stimulation of cDNT in vitro

Converted DNT and *OX40* KO DNT (2 × 10^5^/well) were re-stimulated with 5 μg/ml anti-CD3 and 2 μg/ml anti-CD28 with or without rmIL-2 (50 ng/ml) in a 96-well flat-bottom culture plates for 3 days. EdU (RiboBio, Guangzhou, China) was added to the plates 12 h before harvest (final concentrations were 50 μM). Cell proliferation was measured via EdU incorporation according to the manufacturer’s instructions (EdU staining kit, RiboBio Corporation, Guangzhou, China).

### Re-stimulation of cDNT in vivo

In all, 5 × 10^6^ converted DNT or *OX40* KO DNT were adoptively transferred into B6D2F1 recipient mice by tail vein injection. IL-2/Fc (1 µg/day) and BrdU (100 µg/day) were administered by intraperitoneal injection. Splenocytes were isolated on day 3, cDNT proliferation was measured via BrdU incorporation according to the manufacturer’s instructions (eBioscience).

### Alamar Blue assay

Converted DNT and *OX40* KO DNT (2 × 10^5^/well) were re-stimulated with 5 μg/ml anti-CD3 and 2 μg/ml anti-CD28 with or without rmIL-2 (50 ng/ml) in 96-well flat-bottom culture plates for 5 days. A 1/10th volume of Alamar Blue^®^ reagent was added directly to cells in culture medium, and they were incubated at 37 °C in a cell culture incubator, protected from direct light. The absorbance of Alamar Blue^®^ at 570 nm was measured using 600 nm as a reference wavelength (normalized to the 600 nm value) at different time points.

### Caspase 3/7 activation

Caspase 3/7 activities were measured using the Caspase-Glo 3/7 Assay (Promega, USA). Briefly, the converted DNT and *OX40* KO DNT (2 × 10^5^/well) were re-stimulated with 5 μg/ml anti-CD3 and 2 μg/ml anti-CD28 with or without rmIL-2 (50 ng/ml) in 96-well flat-bottom culture plates for 24, 48, and 72 h. The caspase 3/7 reagent was then added to each well at a different time point, and the plate was incubated on a rotary shaker for 30 min at room temperature. Luminescence was recorded for each well. The caspase 3/7 activity is presented as the mean of the results from the three experiments.

### Quantitative real-time PCR

Total RNA was extracted from cells using an RNeasy mini-kit (Qiagen, Valencia, CA, USA) and reverse transcribed into cDNA with the SuperScript III RT-kit (Invitrogen, Carlsbad, CA, USA). Real-time quantitative polymerase chain reaction was performed with the Power SYBR Green master mix (Applied Biosystems, Foster City, CA, USA) and gene amplification was performed on the ABI 7500 Sequence Detection System (Applied Biosystems). Gene-specific primers used for specific genes and GAPDH are shown in Supplementary Table 1. The relative gene expression was quantitatively analyzed by the comparative Ct method (2^−ΔΔCT^). Data were normalized to the levels of GAPDH mRNA.

### Western blot

Nuclear protein extracts from B6 cDNT and *OX40* KO cDNT were prepared with NE-PER^®^ Nuclear and Cytoplasmic Extraction reagents (Thermo Fisher Scientific, Inc., Pittsburgh, PA, USA). After determination of concentration, the samples containing 20 μg protein were separated by 12% sodium dodecyl sulfate-polyacrylamide gel electrophoresis and transferred to a polyvinylidene difluoride membrane (Millipore). Primary antibodies against p50/p105 (diluted 1:2000), p65 (RelA, diluted 1:2000), and Histone H3 (diluted 1:5000) were used. The membranes were incubated with secondary antibodies conjugated to horseradish peroxidase (HRP), and the proteins were detected by enhanced chemiluminescence (Thermo). The relative density of the protein bands was quantitatively determined using ImageJ software (National Institutes of Health, Bethesda, MD, USA).

### Promoter-binding TF profiling array assay

To examine the potential TFs that bind to the *OX40* core promoter region (−1032/+28 region) in cDNT, TF activation profiling plate array kits were used (Signosis, CA, USA). The assay was performed according to the kit’s instructions. First, 15 μl TF-binding buffer, 3 μl TF probe, 10 μg cDNT nuclear extract, and 5 μl *OX40* promoter PCR fragment were mixed in a tube, and incubated at 20–23 °C for 30 min to form TF/DNA complex. Separation of TF/DNA complex from free probes. Then eluted the bound probe from the complex, and incubated the probe with hybridization plate overnight at 42 °C. After washing with hybridization wash buffer, streptavidin-HRP conjugate were added to each well, which contained the bound probe, and incubated for 45 min gently shaking at room temperature. Finally, incubated with substrate solution for 1 min and detected the probe relative light units on a microplate luminometer (Wallac 1450, Wallac, MA, USA).

### Chromatin immunoprecipitation assay

The chromatin immunoprecipitation (ChIP) assay was performed according to the kit’s instructions (Millipore). After treatment with mouse IL-2 (50 ng/ml) for 48 h, the cDNT were fixed, sonicated, and collected for ChIP assay. DNA fragments were immunoprecipitated with antibodies specific to PPARα, PPARγ, or control rabbit IgG at 4 °C overnight. Subsequently, the DNA fragments were de-crosslinked, purified from the complexes, and ethanol-precipitated. The immunoprecipitated chromatin was amplified by primers (Supplementary Table 1) corresponding to specific regions of the *OX40* genomic locus. Meanwhile, the immunoprecipitated DNA fragments were detected by quantitative real-time. To calculate the fold enrichment of the precipitated PPAR element, each sample was normalized to the corresponding input. All ChIP assays were performed in triplicate.

### Flow cytometric analysis

All samples were acquired on a FACS Aria II flow cytometer (BD Biosciences), and the data were analyzed using FlowJo software (TreeStar, Ashland, OR, USA).

### Statistical analysis

Statistical analysis was performed using the Prism 5.0 software (GraphPad Software, San Diego, CA, USA). The values are expressed as the mean ± SD. Analyses for significant differences were performed using Student’s *t* test and one-way analysis of variance. *p* Values < 0.05 were considered significant.

## Electronic supplementary material

Supplementary Figure 1

Supplementary Figure 2

Supplementary Figure legends
