# Supplementary figures and images for: Critical role of OX40 in the expansion and survival of CD4 T-cell-derived double-negative T cells
Source: Cell Death Dis. 2018 May 23;9(6):616. doi: 10.1038/s41419-018-0659-x (PMC5966453; doi:10.1038/s41419-018-0659-x)

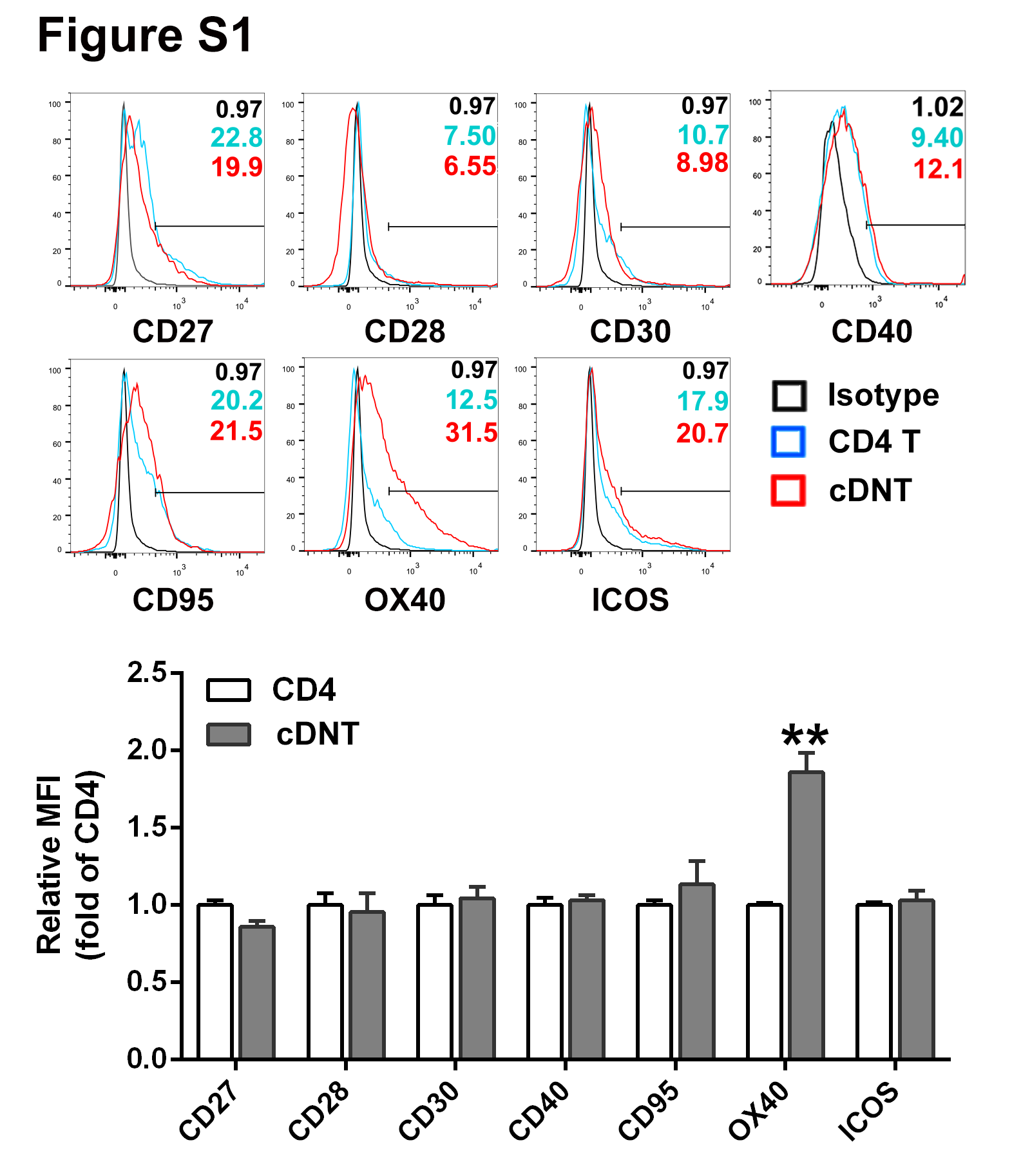

Supplement: Supplementary file 1 — Supplementary Figure 1 [file 41419_2018_659_MOESM1_ESM.tif]

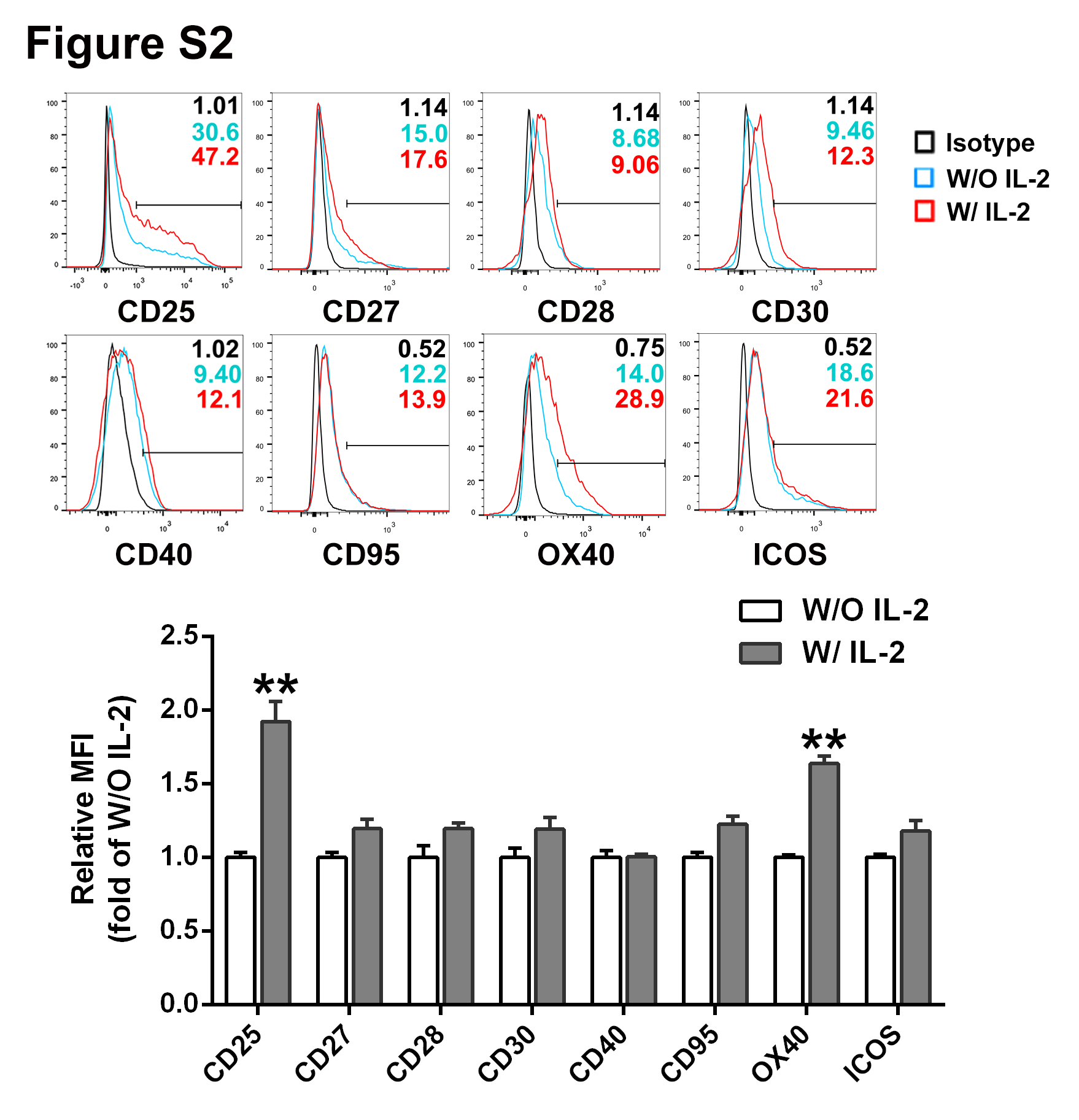

Supplement: Supplementary file 2 — Supplementary Figure 2 [file 41419_2018_659_MOESM2_ESM.tif]
