# Supplementary material for: Critical role of OX40 in the expansion and survival of CD4 T-cell-derived double-negative T cells
Source: Cell Death Dis. 2018 May 23;9(6):616. doi: 10.1038/s41419-018-0659-x (PMC5966453; doi:10.1038/s41419-018-0659-x)
Supplement: Supplementary file 3 — Supplementary Figure legends [file 41419_2018_659_MOESM3_ESM.docx]

**Figure S1 The cell surface markers on cDNT cells and CD4^+^ T cells.**

The induced cDNT cells and CD4^+^ T cells were stained with antibodies to indicate cell-surface markers. The induced cDNT highly expressed OX40, whereas no highly expression of CD27, CD28, CD30, CD40, CD95 and ICOS was observed. Mouse isotype Ig used as control. The data are presented as the mean ± SD, n = 5 in each group. **p*<0.05, ***p*<0.01, NS, no significance.

**Figure S2 Expression of cell-surface markers on sorted cDNT cells after stimulation with IL-2 and anti-CD3/CD28 for 48 h.**

The converted cDNT cells were sorted and stimulated with IL-2 or without IL-2 for 48 h. The cell surface markers, such as CD25, CD28 and other TNFRSF members, were stained. Statistical analysis of the expression on cDNT cells with or without IL-2 based on flow cytometric analysis were shown. The data are presented as the mean ± SD, n = 5 in each group. **p*<0.05, ***p*<0.01, NS, no significance.

| **Supplementary Table 1. Primer sequences used for real-time PCR** | | | | | |
| --- | --- | --- | --- | --- | --- |
| Gene |  | Strand |  | Primer sequence (5'-3') | |
| *OX40* |  | Sense |  | GGGCAGGGAACACAGTCAAC |  |
|  |  | Antisense |  | CAGAATTGCACACCTACTCAG | |
| *Bcl-2* |  | Sense |  | GGAAGGTAGTGTGTGTGG |  |
|  |  | Antisense |  | ACTCCACTCTCTGGGTTCTTGG | |
| *Bcl-xl* |  | Sense |  | AACATCCCAGCTTCACATAACCCC | |
|  |  | Antisense |  | GCGACCCCAGTTTACTCCATCC | |
| *Survivin* |  | Sense |  | GCGGAGGTTGTGGTGAC |  |
|  |  | Antisense |  | AGGGCAGTGGATGAAGC |  |
| *Bcl2l11* |  | Sense |  | GAGATACGGATTGCACAGGA |  |
|  |  | Antisense |  | ATTTGAGGGTGGTCTTCAGC |  |
| *Nfkb1* |  | Sense |  | GTGACAGTGGTGTGGAGACATC | |
|  |  | Antisense |  | GGGGCATTTTGTTCAGAGATAG | |
| *Rela* |  | Sense |  | GGATGGCTACTATGAGGCTGAC | |
|  |  | Antisense |  | AGGTCTCGCTTCTTCACACACT | |
| *Ppara* |  | Sense |  | AGAGCCCCATCTGTCCTCTC |  |
|  |  | Antisense |  | ACTGGTAGTCTGCAAAACCAAA | |
| *Pparg* |  | Sense |  | TGTGGGGATAAAGCATCAGGC | |
|  |  | Antisense |  | CCGGCAGTTAAGATCACACCTAT | |
| *Myc* |  | Sense |  | TCTCCACTCACCAGCACAACTACG | |
|  |  | Antisense |  | ATCTGCTTCAGGACCCT |  |
| *Stat1* |  | Sense |  | TGGAGGAATGTTTCTGTC |  |
|  |  | Antisense |  | GTAAGGAGCACGGTTGT |  |
| *Gata* |  | Sense |  | GGGTTCGGATGTAAGTCGAG |  |
|  |  | Antisense |  | CCACAGTGGGGTAGAGGTTG |  |
| *SP1* |  | Sense |  | AAAGCATATCAAGACTCACCAGAAC | |
|  |  | Antisense |  | ATATTGGTGGTAATAAGGGCTGAA | |
| *Nfat1* |  | Sense |  | CCTGAACGCTGCTGTATG |  |
|  |  | Antisense |  | AACGCTGTTGGAAGAAGAA |  |
| *GAPDH* |  | Sense |  | AAGGTCATCCCAGAGCTGAA |  |
|  |  | Antisense |  | CTGCTTCACCACCTTCTTGA |  |
| *-435/-139* |  | Sense |  | CCCAGTGGTAAGAGCA |  |
|  |  | Antisense |  | CAGAGTCCCATGATAAGC |  |
| *-945/-736* |  | Sense |  | TGGTCCTCAGTATCAACAC |  |
|  |  | Antisense |  | TTGCCCAGGCTAACA |  |
| *-1017/-950* | | Sense |  | AGGCAGGAAGATGGG |  |
|  |  | Antisense |  | TGGGTTGTGGCAAGTAG |  |
